# Deciphering deterministic factors of predation pressures in deep time

**DOI:** 10.1038/s41598-018-35505-1

**Published:** 2018-12-03

**Authors:** Makiko Ishikawa, Tomoki Kase, Hidekazu Tsutsui

**Affiliations:** 1 Department of Earth and Planetary Science, Graduate School of Science, The University of Tokyo, Hongo, Tokyo 113-0033 Japan; 2 Yamazaki University of Animal Health Technology, Hachiouji, Tokyo 192-0364 Japan; 3 Department of Geology and Paleontology, National Museum of Nature and Science, Tsukuba, Ibaraki 305-0005 Japan; 4 Department of Biological Sciences, Kanagawa University, Hiratsuka, Kanagawa 259-1293 Japan; 5 Department of Material Science, Japan Advanced Institute of Science and Technology, Nomi, Ishikawa 923-1211 Japan

## Abstract

Predation pressure occurs as a result of predation frequency and prey vulnerability. Although quantifying these factors individually is essential to precisely understand predation effects on evolution, they have been generally less accessible. Here, using a modified form of Poisson function, we quantified the frequencies and vulnerabilities, as well as the resulting predation pressures, concerning the shell drillers versus prey interactions from the Eocene and Miocene periods. Our analysis quantitatively revealed that low-spired shells tend to show increased vulnerability except for two planispiral species that exhibit an unexpectedly low vulnerability. We then identified septal structures within the two species that resemble those in nautiloids and ammonoids but which provided a defensive role against the predators, enhancing the mean lifetime by approximately 20%. The current approach enables us to quantitatively trace how predation frequency and prey vulnerability have interacted, been transformed spatio-temporally, and been a driving force of evolution at geological time scales.

## Introduction

Predation pressure, which is a major agent for Darwinian natural selection, is associated with two key factors, predation frequency and prey vulnerability^1–5^. Predation involves three principal stages: recognition, catching, and subjugation; correspondingly, from the prey’s perspective: recognition, escape, and resistance. The adaptation to the evolutionary effects of predation often moderate these factors through the three stages. For example, camouflage of leaf insects helps to decrease recognition by predators resulting in a decrease in frequency of predation attempts^6^, whereas the armadillo’s armor makes prey less vulnerable to the subjugation stage of predatory attack^7^. Quantification of such attributes can therefore be essential to understand predation pressure, but this is mostly hampered by the lack of precise predation event histories^8^.

In this study, we show that such quantification can be documented in one form of predator-prey interaction in which predation attempts occur in a discrete manner and are logged onto hard skeletons of prey. The best examples are seen in mollusks, which we focused on in this study.

**Figure 1:**
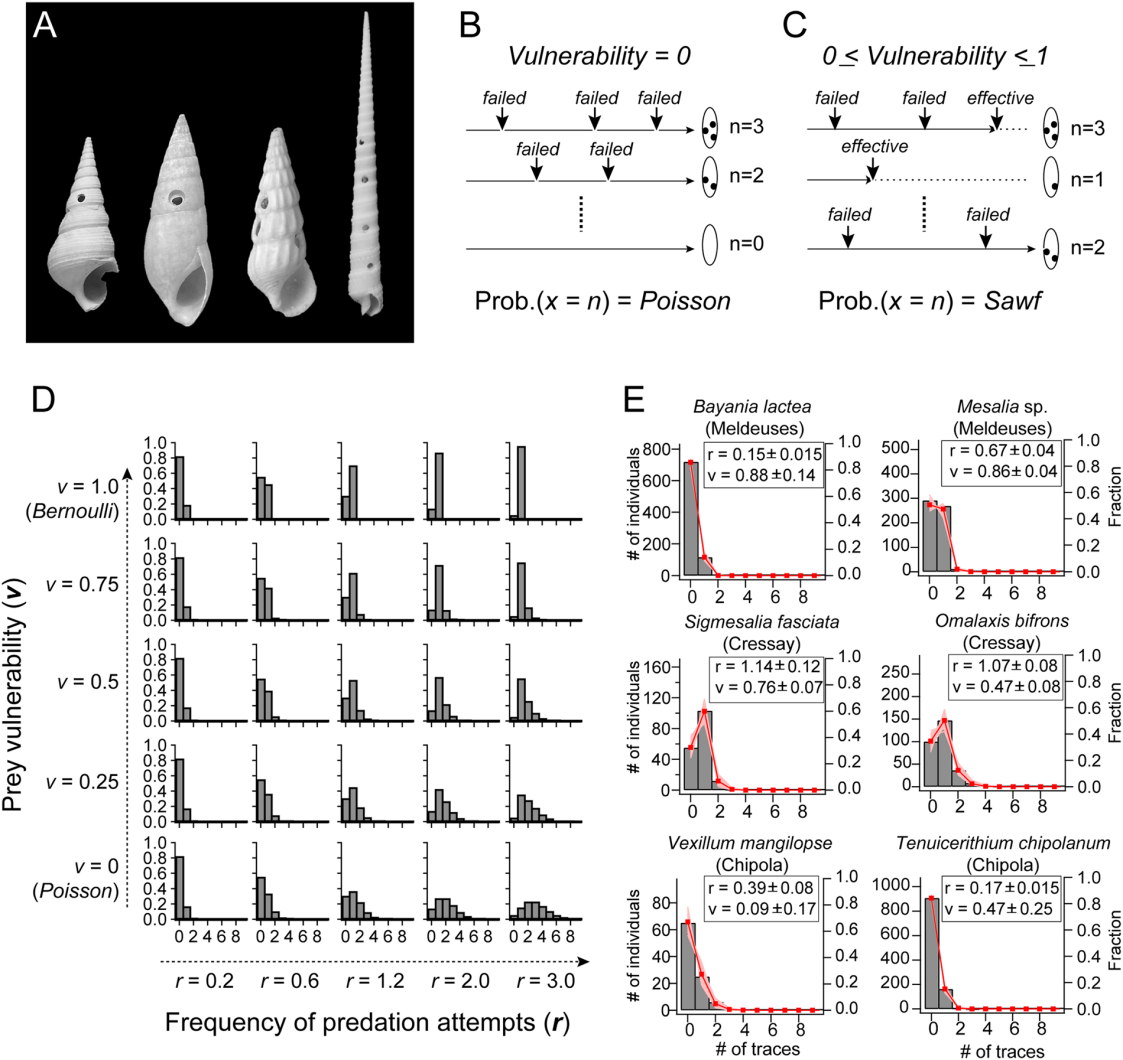
Modeling the formation of multiple drill holes. (**A**) Examples of the holes bored by predatory gastropods. From left: *Mesalia* sp. collected at Iles-les-Meldeuses. Shell height = 26.1 mm. *Bayania lactea* collected at Iles-les-Meldeuses. Shell height = 25.6 mm. *Tenuicerithium chipolanum* collected from Chipola Formation. Shell height = 10.1 mm. The three holes reflect at least two failed predation attempts. *Terebra triseriata* collected off Cebu Island, the Philippines. Shell height = 77.1 mm. (**B**,**C**) Schemes showing logging processes of predation traces. The profile of the expected hole numbers is given by Poisson distribution for null vulnerability (**B**) and by *Sawf function* for general vulnerabilities (**C**). Dotted line indicates lifetime attenuation by effective predations. (**D**) Examples of *Sawf* distribution profiles for 25 different sets of frequency and vulnerability. (**E**) Six examples of observed numbers of predatory traces (in bars) and the results of fits (in lines). Pink lines indicate fits for bootstrap samples (100 traces for each species). The thick red lines indicate the averages of fits (1000 bootstrap samples). The insets show results of quantifications (mean ± S.D.).

Naticids (moon snails), a large family of predatory gastropods, consume the soft body of molluscan prey by drilling a neat circular hole into the shells (Fig. 1A). Shells with hole(s) are commonly found in modern and fossil shell assemblages since the late Cretaceous^9,10^. As the live shells are subject to drilling^11^, traces of multiple drill-holes reflect at least one survival event, since otherwise the number of holes would be one or zero^2,12,13^. This distribution from empty shells thus contains information both on frequency and vulnerability. There have been several studies to interpret the number of drilled holes based on some numerical indices, such as the fraction of shells bearing drill hole(s) and the mean number of holes^2,13–21^. While these indices certainly reflect some aspects of predation, they do not provide direct measures of the two factors of predation individually. In particular, both “high predation frequency” (i.e., high predation pressure) and “low prey vulnerability” (i.e., low predation pressure) tend to increase the expected number of predatory traces. Here, we decipher direct measures of these factors for quantitative and explicit evaluation of predation pressures.

## Results

### Probability distribution for stochastic predation attempts with failures

The predation frequency (***r***) and prey vulnerability (***v***) are defined as the number of predation attempts expected within the intrinsic lifetime and the probability that a single attempt results in a complete predation, respectively. In the special case of ***v*** = 0, the probability that a prey encounters *x* number of predation attempts through the lifetime is given by a Poisson distribution with a mean of ***r*** (Fig. 1B). In general case of 0 ≤ ***v*** ≤ 1, because prey lifetime could be shortened by successful predation, the distribution is altered from Poissonian. We determined that the probability for *x* number of predation attempts is:

1a $$\begin{array}{cc}Sawf(x,r,v)=\frac{{e}^{-r}{r}^{x}{(1-v)}^{x}}{x!}+\frac{\gamma (x,r)}{\Gamma (x)}{(1-v)}^{x-1}v; & {\rm{for}}\,x=1,2,3\ldots \end{array}$$

1b $$\begin{array}{cc}Sawf(x,r,v)={e}^{-r} & {\rm{for}}\,x=0,\end{array}$$

where Γ(*x*) and γ(*x*,*r*) are complete and incomplete gamma functions: ${\rm{\Gamma }}(x)={\int }_{0}^{\infty }{t}^{x-1}{e}^{-t}\,dt$, $\gamma (x,r)={\int }_{0}^{r}{t}^{x-1}{e}^{-t}\,dt$ (Fig. 1C). We here term this distribution as *Sawf* (*Stochastic attempts with failures*) distribution. *Sawf* distribution smoothly connects to Poisson and Bernoulli distributions at ***v*** = **0** and ***1***, respectively (Figs 1D and S1). When ***r*** and ***v*** are given, various aspects of predation pressure can be explicitly evaluated. Thus, for example, the expected fraction of prey surviving all the predation attempts (*Fs*; *fraction of survivors*), and the normalized lifetime attenuated by predations (*Alt*; *attenuated lifetime*) are analytically given by:

2 $$Fs(r,v)={e}^{-rv},$$

and

3 $$Alt(r,v)=\frac{1-{e}^{-rv}}{rv}$$

respectively. Notably, these effects are simple functions of ***r*** × ***v*** (Fig. S2). When *r* or *v* = *0*, *Fs* and *Alt* are both equal to 1, confirming the null effects of predations.

### Fossil data analysis

**Figure 2:**
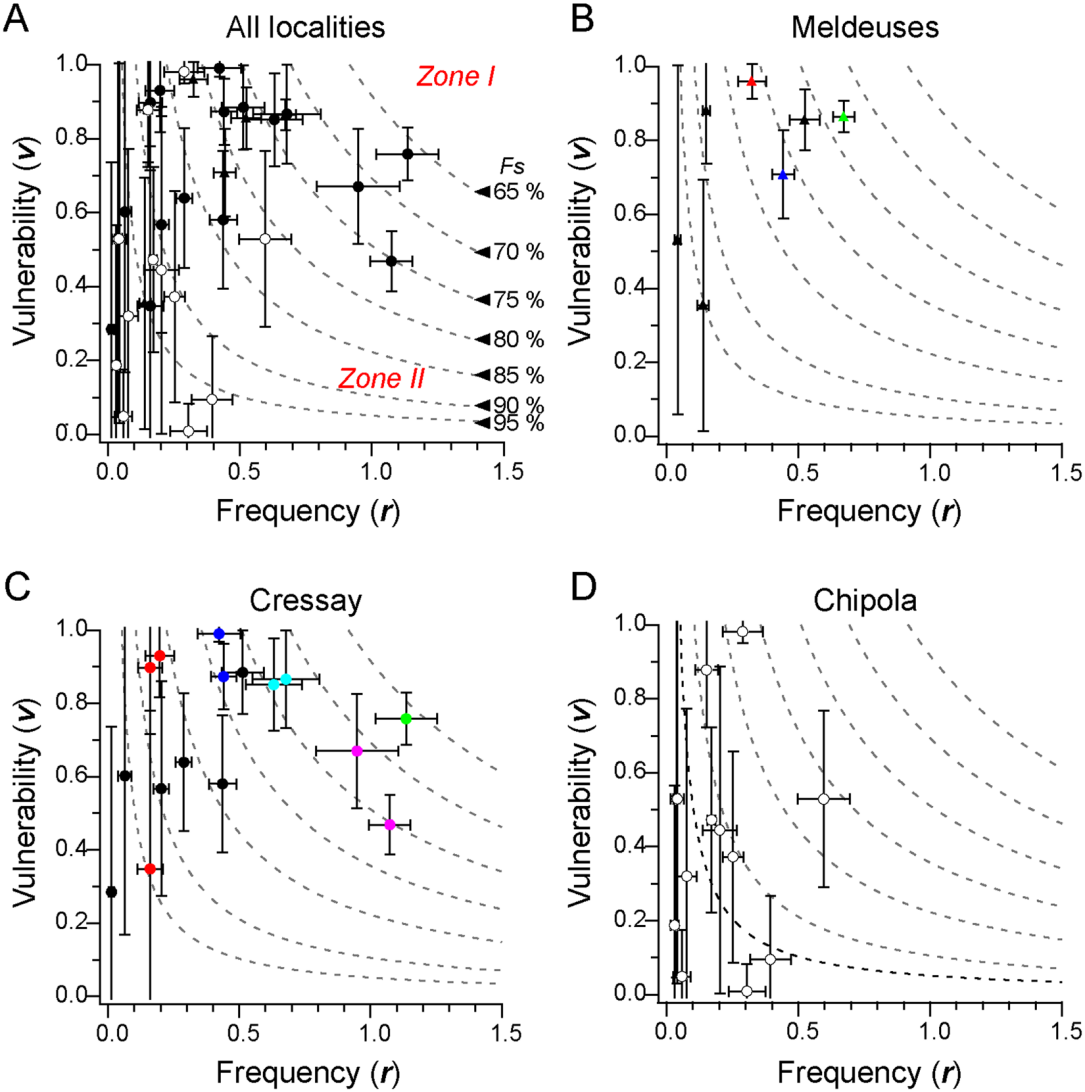
Distributions of predation frequencies and prey vulnerabilities. Scattered plots of the predation frequency versus prey vulnerability for the data in all localities (**A**), and for the species collected in Iles-les-Meldeuses (filled triangles; **B**), Cressay (filled circles; **C**) and Chipola (open circles; **D**). The dotted lines in these plots indicate contours for the expected fraction of survivors (*Fs*; Fig. S2) as the values shown in (**A**). Taxon and symbol colors: Naticidae (red), Ampullinidae (blue), *Keilostoma* (cyan), *Omalaxis* (purple), *Mesalia* and *Sigmesalia* (green).

We analyzed the distributions of numbers of predatory traces in 9,139 gastropod specimens belonging to 39 species, which were collected from middle Eocene beds in Isle-les Meldeuses and Cressay in the Paris Basin, and the lower Miocene Chipola Formation in Florida. Fossil shells from these beds have been the subjects of paleoecological studies on gastropod drilling predation, taking advantage of the excellent preservation of the fossils^22,23^. Although *Sawf* function has only two independent parameters, it well fitted most of the fossil data studied (36 out of 39 species examined; Fig. 1E and Table S1). Figure 2A shows a scatter plot of frequency versus vulnerability for the 36 species. As an overall feature, there exist two blank zones. Zone I covers the region of high frequency and high vulnerability (***r*** > 1; ***v*** > 0.8), whereas Zone II covers that of high frequency and low vulnerability (***r*** ~ 1; ***v*** < 0.2). A possible interpretation of this distribution pattern is that in Zone I the high predation pressure restricts continuous existence of species, whereas in Zone II prey preferences of the predators tend to be biased due to the low-return against drilling costs. In this way, the distribution seems to reflect balance between the two counteracting effects. From the summaries for the three assemblages (Fig. 2B–D), we confirm that the species of the same family in the same assemblage tends to exhibit similar values. One example is a naticid as prey (Cressay, Fig. 2C; red). The predation attempts on naticids were effective with ***v*** > 0.85 except for *Natica* sp.3 from Cressay, which had a large standard deviation. Also, two species of *Keilostoma* (Fig. 2C; cyan) and *Omalaxis bifrons* (Fig. 2C; purple) from Cressay, respectively, exhibited similar frequencies and vulnerabilities. These results are anticipated and also illustrate stability of the quantification procedure. Furthermore, comparisons of the predation frequencies and vulnerabilities in closely related species between the different localities may highlight environmentally specific features, such as density and ecology of predators. For example, naticids (Fig. 2B; red) and ampullinids (Fig. 2B; blue) from Isles-les-Meldeuses, respectively, exhibited frequencies and vulnerabilities similar to those in Cressay (Fig. 2C; red, blue), indicating comparable predation traits. By contrast, predation frequency in *Sigmesalia fasciata* from Cressay (Fig. 2C; green) was almost twice as large as *Mesalia* sp. from Isles-les-Meldueses (Fig. 2B; green), suggesting distinct modes of predation on these related species at these two collecting sites.

### Shell morphology and vulnerability

**Figure 3:**
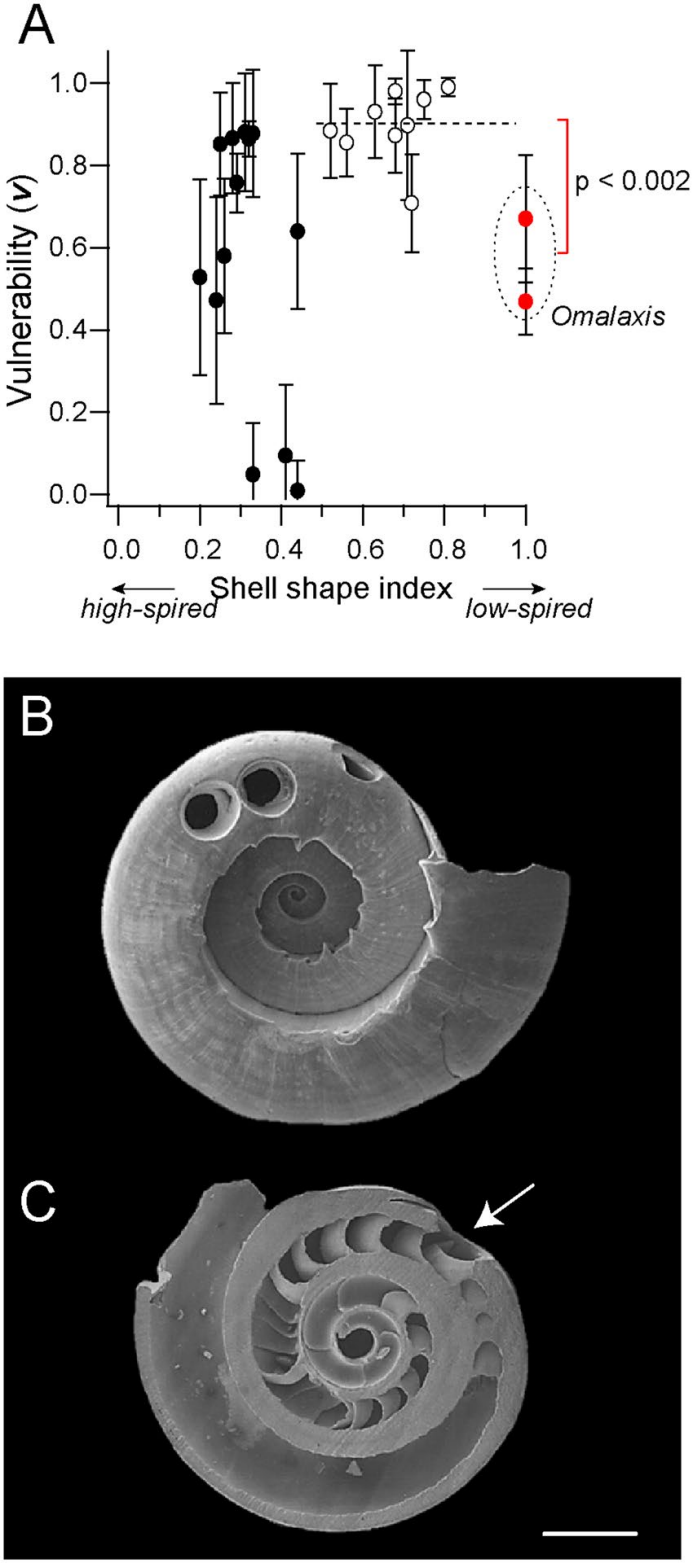
Defensive septa in the Eocene planispiral gastropod *Omalaxis bifrons*. (**A**) A scattered plot of vulnerability versus shell-shape index for 24 species. Species with standard deviations for vulnerability of less than 0.25 were used for analysis. Data for shell-shape index >0.5 are shown in open circles, except for two species of *Omalaxis* shown by red circles. The vulnerabilities for *Omalaxis* were significantly smaller than those for the others (p < 0.002, Wilcoxon Rank-sum test). (**B**) A scanning electron micrograph of *Omalaxis bifrons* collected at Cressay. (**C**) The internal septal structures in *Omalaxis bifrons*. The arrow indicates a trace of predation attempt hampered by one of the septa. Bar = 1 mm (for b, c).

It has long been argued that there exists a correlation between the shell morphology and the vulnerability to predators in marine gastropods^24^. In particular, the “Mesozoic Marine Revolution” hypothesis proposes that the rapid co-evolution between predators and their prey, was boosted by the increased predator-prey interactions from the mid-Mesozoic onward^2,24,25^. During this period, vulnerable traits such as planispiral and trochiform shells were replaced with morphologically more resistant traits, including elongated shells, narrowed and thickened apertures, and elaborated sculptures such as spines. We analyzed vulnerabilities of 24 species that had a moderately small standard deviation (S.D. ≤0.25) along with this hypothesis. The shell-shape index was defined as the aperture height/shell height ratio (Table S1). A plot of vulnerability versus the shell-shape index generally confirmed the previously appreciated trend: high-spired shells generally showed reduced vulnerability (Fig. 3A). However, the two species with low-spired shape exhibited an unexpectedly lower vulnerability based on these indices (Fig. 3A; *dotted circles*). These species correspond to the small planispiral gastropods *Omalaxis bifrons* and *Omalaxis marginata* collected from Eocene beds (Fig. 3B). In order to reveal possible causes for this deviation from the overall trend, we carefully sectioned the shells and closely investigated the internal structures. We then identified numerous septal structures by which the shell is compartmentalized into many small chambers (Fig. 3C). The septa resemble those seen in the nautiloids and ammonoids in which the chambers function to maintain neutral buoyancy by empting/filling cameral liquids through a siphuncle^26^. *Omalaxis* does not have the siphuncle, and the predation from naticids by itself thus reveals the benthic ecology of the species. Our hypothesis is that the septa provide defensive role against predation attempts of shell drillers by restricting the soft parts only to the last whorl. In fact, the micrograph in Fig. 3C shows a trace of an attempt hampered by the septa. The expected fraction of survivors (*Fs*; Eq. 2) in *Omalaxis bifrons* and *O*. *marginata* are 0.6 and 0.53, respectively. Also, the normalized lifetime in these species are 0.79 and 0.74, respectively (*Alt*; Eq. 3). We hypothesized that, from the regression line of vulnerabilities in the other species, gastropods with *Omalaxis*-like overall shell shape but without the internal septal structures, could have vulnerability of ~0.9 and a frequency comparable to that of *Omalaxis* (~1.0). Under these assumptions, *Fs* and *Alt* become 0.41 and 0.66, respectively. Thus, in our estimation, the septal structure enhanced the fraction of survivors and the mean lifetime by 25~50% and 18~20%, respectively.

## Discussion

In three of 39 species analyzed, the observed distributions of predatory traces did not fit well with a single *Sawf* distribution (P-value < 0.05; Table S1). There are some possibilities that account for deviations from the model. First, as a general premise in this type of analysis, data of a given prey species must reflect spatiotemporally consistent interactions with predators throughout. Otherwise the resulting distribution becomes a linear sum of *Sawf* functions with different sets of *r* and *v* parameters. Second, the *Sawf* distribution assumes a simple interaction such as random collisions between a given prey species and predators. More complicated interactions^12,27–30^ would alter the profile. Although these interactions may be also modeled by incorporating additional parameters, significantly larger numbers of samples would be needed to determine the parameters.

In our model, the parameter ***r*** relates to the first two stages (i.e., recognition and catching the prey) among the major three stages of predation. Its definition is equivalent to the expected number of occurrences of the first two stages during the lifetime in the absence of predation of interest. High value of ***r*** indicates situations, for instance, where predator density is high or prey have fewer abilities to avoid the predators. Thus ***r*** reflects multiple aspects including ability to escape, camouflage, predator density, and so on. For example, if one finds species with low ***r*** value even from an environment of high predator density, some adaptation mechanisms to moderate the first two stages might be expected. Also, incorporating the approaches to analyze prey preferences of predators^18,27,31^ may help to address contributions of such aspects to ***r***. On the other hand, the parameter ***v*** reflects resistance in the final subjugation stage. With these clues, in this study, the internal septa structures providing a defensive role was revealed from the Eocene gastropod *Omalaxis*.

The original function of the *Omalaxis* septa structure remains unknown at present. Given that many Palaeozoic gastropods also have septa^32^ and that drilling predation by naticids or muricids increased significantly during the Mesozoic^10,24^, the septa might have an original function other than defense.

In this study, we thus showed that analysis of discrete predatory trace records based on a *Sawf* distribution permits us to quantify the two deterministic factors of predation pressure. While the naticid-gastropod interaction studied here provides rich data on modern and past predation pressures, this mode of interaction appears in various animal and plant taxa^27,28,33–37^. We believe that quantitative knowledge of predator-prey interactions will help ecologists and paleontologists toward a deeper understanding of predation as a driving force of evolution.

## Methods

### Fossil samples

We examined 9,139 fossil gastropod specimens belonging to 39 species in total. They consist of 3,517 individuals belonging to eight species from the middle Eocene (Lutetian) Cressay, Yvelines, Neauphle-le-Vieux, Paris Basin, France; 3,023 individuals belonging to 18 species from the middle Eocene (Bartonian) Isles-les-Meldeuses, Seine et Marne, Paris Basin, France; and 2,599 individuals belonging to 13 species from the lower Miocene Chipola Formation from Florida, USA. Based on criteria used in previous studies^38,39^, the complete predatory naticid bore holes were identified and counted under a dissecting microscope. A scanning electron microscope (T330A, JOEL, Tokyo, Japan) was used to make micrographs of samples after ultrasonic cleaning and gold sputtering. The internal anatomy of *Omalaxis* was investigated after grinding the specimen to the middle line using polishing powder on a glass slide. All the specimens examined in this paper are deposited in the Department of Geology and Paleontology, the National Museum of Nature and Science, Tsukuba, Japan.

### Data analysis

Derivations of the *Sawf*, *Fs*, and *Alt* functions (Eqs 1–3) are described in the supplementary materials and methods. The fitting of count data with *Sawf* function was performed using a custom-made code of MATLAB (MathWorks, MA) on a standard PC. The best fit parameters for the predation frequency (***r***) and prey vulnerability (***v***) as well as their standard errors, were determined by least squares and bootstrap (n = 1000) methods, respectively. Goodness of Fit (P-value) was evaluated using a “multinomial.test” function of R software (www.r-project.org) in CX250 Cluster. The Monte-Carlo method of 10^7^ trials was used for these evaluations. Figure S3 shows sample code for fitting with *Sawf* function.

## Electronic supplementary material

Supplementary information

## Data Availability

All the specimens examined in this paper are deposited in the Department of Geology and Paleontology, the National Museum of Nature and Science, Tsukuba, Japan.
