# Supplementary material for: Deciphering deterministic factors of predation pressures in deep time
Source: Sci Rep. 2018 Dec 3;8:17532. doi: 10.1038/s41598-018-35505-1 (PMC6277388; doi:10.1038/s41598-018-35505-1)
Supplement: Supplementary file 1 — Supplementary information [file 41598_2018_35505_MOESM1_ESM.pdf]

Supplementary Information for

Deciphering deterministic factors of predation pressures in deep time

Makiko Ishikawa[1,2]*, Tomoki Kase[3,4], Hidekazu Tsutsui[5]

[1] Department of Earth and Planetary Science, Graduate School of Science, The University of Tokyo, Hongo, Tokyo 113-0033, Japan.
[2] Yamazaki University of Animal Health Technology, Hachiouji, Tokyo 192-0364, Japan.
[3] Department of Geology and Paleontology, National Museum of Nature and Science, Tsukuba, Ibaraki 305-0005, Japan.
[4] Department of Biological Sciences, Kanagawa University, Hiratsuka, Kanagawa 259-1293, Japan.
[5] Department of Material Science, Japan Advanced Institute of Science and Technology, Nomi, Ishikawa 923-1211, Japan.

*Correspondence to: Makiko Ishikawa, Department of Earth and Planetary Science, Graduate School of Science, The University of Tokyo.
Email: makiko@eps.s.u-tokyo.ac.jp

Formulation

Sawf distribution is derived as follows. We define $\boldsymbol{L}$ as an intrinsic lifetime of prey in the absence of predators of interest (but in the presence of all other environmental stress including the other kinds of predators), and the predation frequency ($\boldsymbol{r}$) as the number of predation attempts expected to encounter in a time period of $\boldsymbol{L}$**.** Therefore, $\boldsymbol{r}$ is dimensionless. This definition, compared to that in actual time (e.g., "*x* attempts per a year"), helps to avoid consideration of complicating factors such as age structure, which are not always definitely known in the fossil record. The prey vulnerability, $\boldsymbol{v}$ ($0 \leq \boldsymbol{v} \leq 1$), is defined as a probability that a single predation attempt results in a complete predation. Our interest is the probability distribution for number of predatory traces left on the prey body as a function of $\boldsymbol{r}$ and $\boldsymbol{v}$. We consider segmentation of $\boldsymbol{L}$ into $\boldsymbol{N}$ time steps (Scheme1).

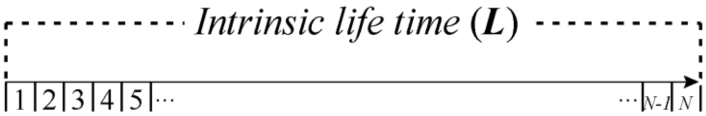

(Scheme 1)

The probability that a prey encounters a predation attempt within a single time step of $\frac{L}{N}$ becomes equal to $\frac{r}{N}$. When considering large $\boldsymbol{N}$ (i.e., sufficiently short time step), the probability for multiple attempts in a single time step is ignored. Here, $\frac{r}{N}v$ and $\frac{r}{N}(1-v)$ respectively, give the probability that effective and ineffective attempts occur within a single time step. First we consider a situation in which a prey survives all predation attempts. The diagram below (Scheme 2) illustlates an example of a prey surviving three attempts. The "*ne*" and "○" indicate occurrence of *no event* and *ineffective* predation attempts, respectively.

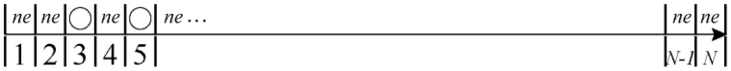

(Scheme 2)

Then, a probability that a prey survives all $\boldsymbol{x}$ predation attempts is written as:

$$Survived = \frac{N!}{x!(N-x)!}\left(1-\frac{r}{N}\right)^{N-x}\left(\frac{r}{N}(1-v)\right)^{x} \qquad ; \text{For } \boldsymbol{x} = 0, 1, 2, 3\ldots \quad (4)$$

By taking limit of $\boldsymbol{N}\rightarrow\infty$, we obtain:

$$\lim_{N\to\infty} Survived = \frac{e^{-r} r^{x}(1-v)^{x}}{x!} \qquad ; \text{For } \boldsymbol{x} = 0, 1, 2, 3\ldots \qquad (5)$$

Next, another situation is considered in which a prey is preyed upon after surviving ***x-1*** predation attempts. The diagram below illustrates three examples for ***x***=*3* (Scheme 3). The dotted line indicates the attenuation of lifetime by an effective predation as indicated by "●".

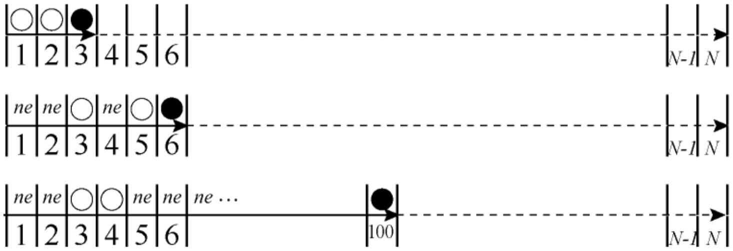

(Scheme 3)

A probability for general ***x*** is written as:

$$Preyed = \left[1+\frac{x!}{(x-1)!}\left(1-\frac{r}{N}\right)+\frac{(x+1)!}{(x-1)!2!}\left(1-\frac{r}{N}\right)^{2}+....+\frac{(N-1)!}{(x-1)!(N-x)!}\left(1-\frac{r}{N}\right)^{N-x}\right]\left(\frac{r}{N}\right)^{x}(1-v)^{x-1}v$$

; For ***x*** = 1, 2, 3, …. (6)

By taking the limit of $\boldsymbol{N}\rightarrow\infty$, we obtain:

$$\lim_{N\to\infty} Preyed = \left[1-e^{-r}\left(1+r+\frac{r^{2}}{2}+...+\frac{r^{x-1}}{(x-1)!}\right)\right](1-v)^{x-1}v$$

; For ***x*** = 1, 2, 3… (7)

The sum of the series (7) is simplified as:

$$Preyed = \frac{\gamma(x,r)}{\Gamma(x)}(1-v)^{x-1}v \qquad ; \text{For } \boldsymbol{x} = 1, 2, 3\ldots \quad (8)$$

where $\Gamma(x)=\int_{0}^{\infty} t^{x-1}e^{-t}dt$ and $\gamma(x,r)=\int_{0}^{r} t^{x-1}e^{-t}dt$.

The two situations considered by the equations (5, 8) are exclusive and both result in ***x*** predatory

traces. A probability distribution of interest here is thus given as the sum:

$$Sawf\,(x,r,v) = Survived + Preyed$$

$$= \begin{cases} \dfrac{e^{-r} r^{x} (1-v)^{x}}{x!} + \dfrac{\gamma(x,r)}{\Gamma(x)} (1-v)^{x-1} v & \text{; For } x = 1,2,3... \\ e^{-r} & \text{; For } x = 0 \end{cases} \qquad (1a,b)$$

The fraction of survivors (*Fs*) is given by integrating the equation (5) for $\boldsymbol{x}$:

$$Fs(r,v) = \sum_{x} Survived = \sum_{x=0}^{\infty} \frac{e^{-r} r^{x} (1-v)^{x}}{x!} = e^{-rv} \qquad (2)$$

The attenuated lifetime (*Alt*) is given by summing the expected lifetime in the two situations, *Survived* and *Preyed*. Again, this gives a simple function of $r \times v$.

$$Alt(r,v) = \frac{L \cdot Fs(r,v) + \underset{N\to\infty}{Lim} \sum_{k=1}^{N} \left[\dfrac{kL}{N} \left(1 - \dfrac{rv}{N} \right)^{k-1} \dfrac{rv}{N} \right]}{L} \qquad (3)$$

$$= \frac{1-e^{-rv}}{rv}$$

Table S1. Fossil data and results of analysis.

	Species	Number of individual(s) with *x* numbers of hole(s)																
		0	***1***	***2***	***3***	***4***	***5***	***≧6***	***total***	*r (mean ± SD)*	*v (mean ± SD)*	P-value[a)]	***Fs*** [b)]	***Alt*** [c)]	*shells with hole(s)* [d)]	*Mean # of holes* [e)]	*shell height (cm)* [f)]	*shell shape index* [g)]	*Fossil locality*	*Geologic age*
1	*Bayania lactea*	718	116	0	0	0	0	0	834	0.15 ± 0.01	0.88 ± 0.14	0.92	0.88	0.94	0.14	0.14	3.2	0.31	Isles-les-Meldeus [h)]	M. Eocene (Bartonian)
2	*Bayania* sp.	396	17	0	0	0	0	0	413	0.04 ± 0.01	0.53 ± 0.47	0.99	0.98	0.99	0.04	0.04	1.1	0.28	Isles-les-Meldeus	M. Eocene (Bartonian)
3	*Olivella* sp.	128	85	3	0	0	0	0	216	0.52 ± 0.06	0.86 ± 0.08	0.98	0.64	0.81	0.41	0.42	1.4	0.56	Isles-les-Meldeus	M. Eocene (Bartonian)
4	*Ampullina parisiensis*	202	105	6	1	0	0	0	314	0.44 ± 0.04	0.71 ± 0.12	0.44	0.73	0.86	0.36	0.38	2.8	0.72	Isles-les-Meldeus	M. Eocene (Bartonian)
5	*Natica epiglottinoides*	95	36	0	0	0	0	0	131	0.32 ± 0.05	0.96 ± 0.05	1.00	0.73	0.86	0.27	0.27	1.3	0.75	Isles-les-Meldeus	M. Eocene (Bartonian)
6	*Serratocerithium serratum*	315	45	2	1	0	0	0	363	0.14 ± 0.02	0.35 ± 0.34	0.11	0.95	0.98	0.13	0.14	4.4	0.25	Isles-les-Meldeus	M. Eocene (Bartonian)
7	*Granulolabium thiarella*	658	16	2	0	0	0	0	676	0.03 ± 0.01	0.28 ± 0.45	0.02	nd	nd	0.03	0.03	2.1	0.30	Isles-les-Meldeus	M. Eocene (Bartonian)
8	*Mesalia* sp.	291	268	11	0	0	0	0	570	0.67 ± 0.04	0.86 ± 0.04	0.98	0.56	0.76	0.49	0.51	2.1	0.32	Isles-les-Meldeus	M. Eocene (Bartonian)
9	*Natica* sp.2	75	13	0	0	0	0	0	88	0.16 ± 0.04	0.90 ± 0.18	1.00	0.87	0.93	0.15	0.15	1.3	0.71	Cressay [i)]	M. Eocene (Lutetian)
10	*Natica* sp.3	62	10	1	0	0	0	0	73	0.16 ± 0.05	0.35 ± 0.43	0.56	0.95	0.97	0.15	0.16	1.0	0.73	Cressay	M. Eocene (Lutetian)
11	*Omalaxis bifrons*	100	147	36	8	0	1	0	292	1.07 ± 0.08	0.47 ± 0.08	0.31	0.60	0.79	0.66	0.85	0.8	1.00	Cressay	M. Eocene (Lutetian)
12	*Omalaxis marginata*	25	34	4	1	1	0	0	65	0.95 ± 0.16	0.67 ± 0.16	0.06	0.53	0.74	0.62	0.75	0.9	1.00	Cressay	M. Eocene (Lutetian)
13	*Tricolia* sp.	103	7	0	0	0	0	0	110	0.06 ± 0.03	0.60 ± 0.43	0.86	0.96	0.98	0.06	0.06	0.6	0.50	Cressay	M. Eocene (Lutetian)
14	*Marginella* cf. *angystoma*	219	47	2	0	0	0	0	268	0.20 ± 0.03	0.57 ± 0.29	0.99	0.89	0.94	0.18	0.19	0.7	0.90	Cressay	M. Eocene (Lutetian)
15	*Cryptoconus lineolatus*	62	40	1	0	0	0	0	103	0.51 ± 0.08	0.88 ± 0.11	1.00	0.64	0.80	0.40	0.41	0.8	0.52	Cressay	M. Eocene (Lutetian)
16	*Sigmesalia fasciata*	55	103	12	1	0	0	0	171	1.14 ± 0.12	0.76 ± 0.07	0.99	0.42	0.67	0.68	0.76	2.9	0.29	Cressay	M. Eocene (Lutetian)
17	*Keilostoma* sp.	29	27	1	0	0	0	0	57	0.68 ± 0.13	0.87 ± 0.13	1.00	0.56	0.76	0.49	0.51	1.8	0.28	Cressay	M. Eocene (Lutetian)
18	*Keilostoma turricula*	40	33	1	1	0	0	0	75	0.63 ± 0.11	0.85 ± 0.13	0.08	0.58	0.77	0.47	0.51	2.0	0.25	Cressay	M. Eocene (Lutetian)
19	Turridae sp.	76	9	3	1	0	0	0	89	0.14 ± 0.04	0.02 ± 0.13	0.01	nd	nd	0.15	0.20	1.5	0.38	Cressay	M. Eocene (Lutetian)
20	*Diastoma costellatum*	119	60	5	1	0	0	0	185	0.44 ± 0.05	0.58 ± 0.19	0.49	0.78	0.88	0.36	0.39	3.0	0.26	Cressay	M. Eocene (Lutetian)
21	*Exechestoma interruptum angulosum*	146	2	0	0	0	0	0	148	0.01 ± 0.01	0.29 ± 0.45	0.72	1.00	1.00	0.01	0.01	1.3	0.26	Cressay	M. Eocene (Lutetian)
22	*Ptychocerithium lamellosum*	406	145	13	3	3	0	0	570	0.34 ± 0.03	0.36 ± 0.16	0.00	nd	nd	0.29	0.34	1.9	0.28	Cressay	M. Eocene (Lutetian)
23	*Natica* sp.4	69	15	0	0	0	0	0	84	0.20 ± 0.05	0.93 ± 0.11	1.00	0.83	0.91	0.18	0.18	1.2	0.63	Cressay	M. Eocene (Lutetian)
24	*Ampullina* sp.	152	82	2	0	0	0	0	236	0.44 ± 0.05	0.87 ± 0.09	1.00	0.68	0.83	0.36	0.36	1.7	0.68	Cressay	M. Eocene (Lutetian)
25	*Cepatia cepacea*	53	28	0	0	0	0	0	81	0.42 ± 0.08	0.99 ± 0.02	1.00	0.66	0.82	0.35	0.35	0.8	0.81	Cressay	M. Eocene (Lutetian)
26	*Trypatrochus conicum*	246	78	4	0	0	0	0	328	0.29 ± 0.03	0.64 ± 0.19	0.98	0.83	0.91	0.25	0.26	0.7	0.44	Cressay	M. Eocene (Lutetian)
27	*Vexillum mangilopse*	65	25	6	1	0	0	0	97	0.39 ± 0.08	0.09 ± 0.17	0.58	0.96	0.98	0.33	0.41	0.9	0.41	Chipola [j)]	Early Miocene
28	*Astrea chipolana*	93	15	0	0	0	0	0	108	0.15 ± 0.04	0.88 ± 0.15	1.00	0.87	0.94	0.14	0.14	1.6	0.33	Chipola	Early Miocene
29	*Triicolia affinis chipolana*	471	18	0	0	0	0	0	489	0.04 ± 0.01	0.53 ± 0.48	0.99	0.98	0.99	0.04	0.04	1.1	0.35	Chipola	Early Miocene
30	*Mitrella* sp.	84	14	5	1	0	0	0	104	0.31 ± 0.07	0.01 ± 0.07	0.07	1.00	1.00	0.19	0.26	1.1	0.24	Chipola	Early Miocene
31	*Tenuicerithium chipolanum*	907	162	7	1	0	0	0	1077	0.17 ± 0.02	0.47 ± 0.25	0.37	0.92	0.96	0.16	0.17	1.3	0.24	Chipola	Early Miocene
32	*Nassarius harrisi*	151	40	3	1	0	0	0	195	0.25 ± 0.04	0.37 ± 0.29	0.29	0.91	0.95	0.23	0.25	0.9	0.39	Chipola	Early Miocene
33	*Crepidula* sp.	147	4	1	0	0	0	0	152	0.03 ± 0.02	0.19 ± 0.38	0.08	0.99	1.00	0.03	0.04	1.4	1.00	Chipola	Early Miocene
34	*Hemicerithium pagodum*	70	3	0	0	0	0	0	73	0.04 ± 0.03	0.53 ± 0.50	0.84	0.98	0.99	0.04	0.04	0.9	0.24	Chipola	Early Miocene
35	*Tenuicerithium* ? sp.	43	30	4	0	0	0	0	77	0.60 ± 0.10	0.53 ± 0.24	1.00	0.73	0.86	0.44	0.49	0.9	0.20	Chipola	Early Miocene
36	*Olivella cotinados*	47	3	1	0	0	0	0	51	0.08 ± 0.04	0.32 ± 0.45	0.14	0.98	0.99	0.08	0.10	1.4	0.59	Chipola	Early Miocene
37	*Marginella nanna*	45	15	0	0	0	0	0	60	0.29 ± 0.08	0.98 ± 0.03	1.00	0.75	0.87	0.25	0.25	0.4	0.68	Chipola	Early Miocene
38	*Calliostoma grammaticum*	54	3	0	0	0	0	0	57	0.06 ± 0.03	0.05 ± 0.13	1.00	1.00	1.00	0.05	0.05	1.3	0.33	Chipola	Early Miocene
39	*Acteocina incisula curtoides*	48	10	1	0	0	0	0	59	0.20 ± 0.06	0.44 ± 0.44	0.61	0.91	0.96	0.19	0.20	0.8	0.86	Chipola	Early Miocene
	total								**9139**											

[a)] data for P-value <0.05 are shown in gray, [b)] Fs; fraction of survivors, [c)] Alt; attenuated lifetime, [d)] Fraction of shells bearing drill hole(s). [e)]Mean number of drill holes in a shell, [f)] Shell height (or shell diameter for *Crepidula* and *Omalaxis*) of the most largest specimen, [g)] Defined as the aperture height / shell height ratio, [h)] Isles-les-Meldeuses; Isles-les-Meldeuses, Seine et Marne, Paris Basin, France, [i)] Cressay; Cressay, Yvelines, Paris Basin, France, [j)] Chipola; Chipola Formation; Florida, USA

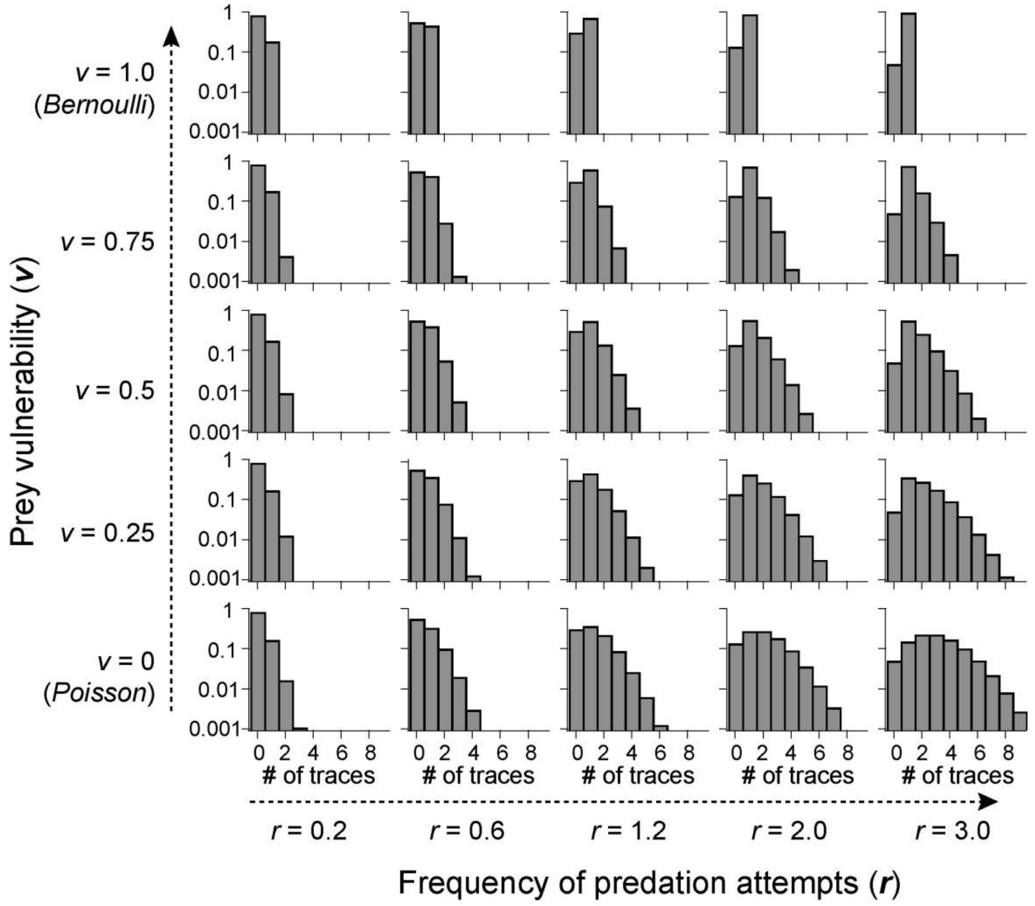

Fig. S1 The profiles of *Sawf* distribution in the log-scale.

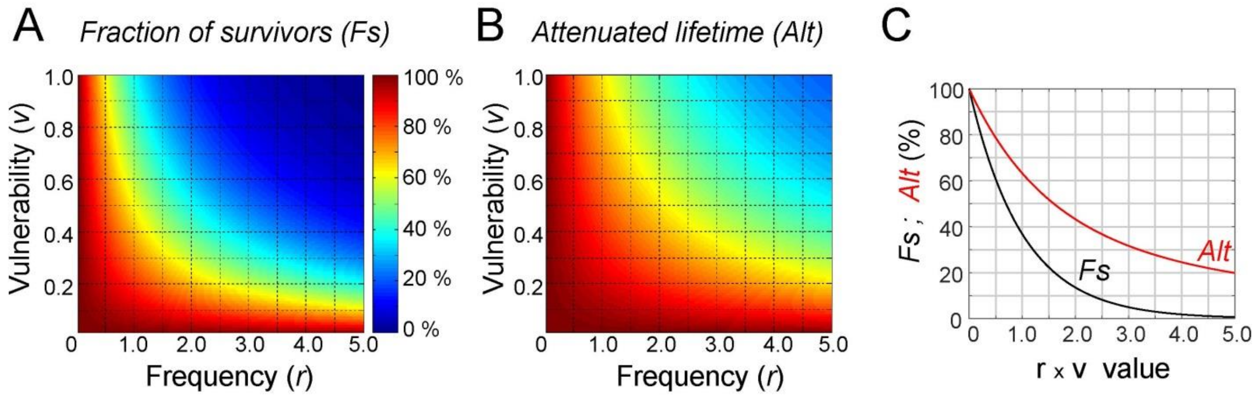

Fig. S2 Predation pressure as a function of frequency (*r*) and vulnerability (*v*).

(A, B) Color-coded maps for the expected fraction of survivors (A; *Fs*) and the attenuated lifetime (B; *Alt*) presented as a function of the predation frequency and vulnerability. The color bar in (A) is also applicable to (B). **(C)** Plot of the *Fs* and *Alt* in [%] as a function of $r \times v$ (i.e., frequency × vulnerability) value.

Fig. S3. Sample MATLAB code for fitting data with a *Sawf* function.

```
data=[202 105 6 1 0 0 0 0 0 0 0]; %input data (# of specimens for 0, 1, 2...10 traces)

gs=201;    % grid resolution for r and v
rmax=2.0;
vmax=1.0;
data=data./sum(data);
    for i=1:gs
        for j=1:gs
            x=[0:10];
            r=(i-1).*rmax./(gs-1);
            v=(j-1).*vmax./(gs-1);
            f=exp(-r).*(r.^x).*(1-v).^x./factorial(x);
            x=[1:10];
            g=gammainc(r,x).*(1-v).^(x-1).*v;
            fg=f+[0 g];
            score(i,j)=sum((data-fg).^2);
        end
    end

    [m,xx]=min(score);
    [m,yy]=min(min(score));

    r=(xx(yy)-1).*rmax./(gs-1);
    v=(yy-1).*vmax./(gs-1);
    x=[0:10];
    f=exp(-r).*(r.^x).*(1-v).^x./factorial(x);
    x=[1:10];
    g=gammainc(r,x).*(1-v).^(x-1).*v;
    fg=f+[0 g];
    data
    PredationFrquency=r
    Vulnerability=v
    fit=fg
    chi2=sum((data-fg).*(data-fg)/fg)
    x=[0:10];
    figure
    plot(x,data,'-ro',x,fit,'-.b')
```
